# Materia Medica and Hygiene in the University of Buffalo

**Published:** 1863-06

**Authors:** 


*Materia Medica and Hygiene in the University of Buffalo.*—We understand that Prof. Charles A. Lee, having completed his European tour and returned to this country, resumes the duties of his Professorship in the University of Buffalo, and will lecture at the approaching term upon Materia Medica and Hygiene.

Lectures upon Materia Medica during the absence of Prof. Lee have been given with unsurpassed ability by Dr. J. R. Lothrop. Hygiene is now added to the duties of this Professorship.

